# Corrosion Resistance of Amorphous Carbon Coatings Doped with Nitrogen and Hydrogen in 3.5% NaCl Solution and Mine Waters

**DOI:** 10.3390/ma18204703

**Published:** 2025-10-14

**Authors:** Karol Wunsch, Tomasz Borowski, Jerzy Robert Sobiecki, Andrzej Wieczorek

**Affiliations:** 1 Faculty of Materials Science and Engineering, Warsaw University of Technology, Wołoska 141, 02-507 Warsaw, Poland; jerzy.sobiecki@pw.edu.pl; 2 Faculty of Mining and Geology, Silesian University of Technology, Akademicka 2A, 44-100 Gliwice, Poland; andrzej.n.wieczorek@polsl.pl

**Keywords:** RFCVD, DLC, corrosion resistance

## Abstract

To evaluate the effect of nitrogen and hydrogen on the corrosion resistance of diamond-like carbon (DLC) coatings, potentiodynamic tests were carried out on DLC coatings deposited under various reactive atmosphere compositions on martensitically hardened 34CrAlNi steel. In order to replicate actual operating conditions of steel components, the tests were conducted both in a reference 3.5% NaCl solution and in natural mine waters, which are in direct contact with mining gearbox mechanisms. Although it is generally assumed that the addition of other elements tends to deteriorate the corrosion resistance of amorphous carbon coatings, such doping simultaneously improves adhesion to metallic substrates, and enhanced adhesion in turn contributes to improved corrosion resistance. Variation in the proportions of the doping elements altered the damage morphology on the sample surface, as well as the corrosion current density, corrosion potential, and polarization resistance. Improvements in corrosion resistance parameters correlated with the quality of the coating–substrate adhesion, evaluated in accordance with the VDI3918 standard. The most favorable properties were obtained for coatings deposited under a gas composition of N_2_:CH_4_:H_2_ with a ratio of 3:4:2.

## 1. Introduction

Carbon-based coatings have been widely studied due to their high hardness [1,2], resistance to wear [2,3,4,5,6], and corrosion [7,8,9,10,11], which make them suitable for a broad range of engineering applications. Such coatings can be produced by various methods, including physical vapor deposition (PVD) from a graphite target [12,13,14,15] and chemical vapor deposition (CVD) [1,16,17,18]. Among CVD techniques, radio frequency CVD (RFCVD) enables deposition of carbon coatings at low temperatures, which is advantageous for substrates sensitive to heat or with limited thermal stability [19,20,21,22].

DLC coatings exhibit high corrosion resistance in diverse environments, owing to their chemical inertness to acids, bases, and organic solvents at room temperature [7,17]. Increasing coating thickness, associated with longer deposition time, enhances their protective properties [23]. The coating serves as a physical barrier that shields the substrate from corrosive agents, while its amorphous, homogeneous structure helps to suppress localized corrosion (e.g., pitting) [8]. Corrosion protection is further improved with stronger adhesion to the substrate [24].

Although a higher sp^3^ bonding fraction—for example, through the choice of carbon coating precursor [25]—reduces charge transfer, which may influence the electrochemical properties of the coating, studies in concentrated nitric acid have not confirmed a direct correlation between corrosion resistance and sp^3^ content [18]. Conversely, an increased fraction of graphite-like phases, achieved by adjusting RFCVD process pressure, has been shown to reduce internal stresses and increase coating density, which positively affects corrosion resistance [26]. Corrosion resistance also depends on surface quality and adhesion, both of which may be compromised by defects arising from improper sample preparation or solid contaminants, particularly in large-scale industrial settings [27]. Such defects occur more frequently in PVD-deposited coatings than in plasma-assisted CVD (PACVD) coatings, where surface cleaning can occur directly before the coating process. In industrial practice, maintaining coating homogeneity presents a particular challenge [27]. Additionally, porosity induced by inappropriate process parameters or precursor gas selection may negatively affect corrosion resistance [25]. Enhancing adhesion, for instance through substrate nitriding, improves corrosion resistance [9,10]. Similar benefits are observed in DLC doping with fluorine or silicon, which not only enhance its corrosion resistance but also reduce internal stresses, thereby improving adhesion and wear resistance [28,29]. However, improper incorporation of dopants, without a corresponding improvement in adhesion, can diminish the corrosion resistance of the coating [11,30,31]. Thus, given the complex interplay between structure, chemical composition, and corrosion resistance of carbon coatings, it is essential to evaluate their performance under conditions that closely replicate the actual operating environment of the treated component.

One effective method of enhancing the adhesion of carbon coatings is the introduction of hydrogen and nitrogen dopants. These are among the most frequently applied and best-studied dopants for DLC coatings [32,33,34], with their widespread use also attributed to the availability and ease of incorporating H_2_ and N_2_ gases in PACVD processes. These elements exert partially opposing effects: Hydrogen (up to a certain concentration [35]) increases the hardness of carbon coatings while simultaneously raising their internal stresses [32,36,37], which may reduce adhesion to metallic substrates [38]. In contrast, nitrogen addition decreases internal stresses, hardness, and Young’s modulus as its concentration within the carbon coating structure increases [4,39,40,41,42,43,44]. The nitrogen content in the coating is linearly dependent on its concentration in the reactive atmosphere, and its incorporation decreases with rising synthesis temperature [40,45,46]. Previous studies have demonstrated that the simultaneous introduction of both gases as dopants can produce a synergistic effect by limiting delamination, improving coating adhesion, and reducing the incorporation of oxygen atoms as impurities within the coating [47]. Such co-doping has been shown to enhance adhesion, which in turn positively influences the tribological properties of carbon coatings. The present study aims to determine whether this type of modification also contributes to improved corrosion resistance, since enhanced adhesion is expected to correlate with superior protective properties in this respect. Since this work continues our previous studies, readers are encouraged to consult [47] for details on the coatings’ compositions, thicknesses, and scratch test adhesion.

## 2. Materials and Methods

This study employed 34CrAlNi steel samples, with their chemical composition presented in Table 1. The samples were prepared in the form of disks with a thickness of 8 mm and a diameter of 25.4 mm, enabling all required tests to be performed on a single specimen. Austenitization was carried out at 900 °C for 30 min, followed by martensitic quenching in water to achieve maximum possible hardness, thereby enhancing coating adhesion. After heat treatment, the samples were ground with 800-grit abrasive paper and subsequently etched by immersion in a 5% Nital solution for 60 s to expand the contact surface.

Carbon coatings were deposited using a Roth & Rau 400 system via the RFCVD process, operating at a supply frequency of 13.56 MHz. Prior to deposition of the functional coating, sputter cleaning was carried out for 6 min in an argon–hydrogen plasma with a 4:1 (Ar:H_2_) ratio at a pressure of 1 × 10^−2^ mbar and a discharge power of 600 W. Coating deposition was then performed under the following conditions: deposition time—30 min, discharge power—600 W, pressure—1 × 10^−2^ mbar. The process temperature increased naturally during treatment and did not exceed 80 °C.

Four process variants were conducted, differing in the proportions of nitrogen, methane, and hydrogen (listed in this order) in the reactive atmosphere: 1:4:1, 4:4:1, 1:4:4, and 3:4:2. The proportion has been controlled by flow of the gases. The flow rates are ten times higher than the given ratio; for example, a 1:4:1 ratio corresponds to flow rates of 10:40:10 sccm for the gases used. These parameters and atmospheric compositions were selected based on earlier studies that enabled the production of carbon coatings with satisfactory adhesion to steel substrates without the need for a transition interlayer and without the risk of structural degradation induced by temperature [21,47]. The coatings obtained in this manner should therefore be directly comparable with those produced in previous investigations [47], allowing reference to their adhesion parameters, chemical compositions, and thicknesses when discussing the present results.

Given the previously highlighted differences in the behavior of carbon coatings under various corrosive conditions, corrosion tests were conducted in both model and application-relevant environments. Accordingly, samples were tested in a reference 3.5% NaCl solution (with a chloride concentration equivalent to 21,231 mg/L) and in mine waters collected from operating mines, with compositions being measured in the Accredited Laboratory of PWiK in Gliwice and being presented in Table 2.

Electrochemical tests were performed in a three-electrode configuration, where the sample served as the working electrode, a Ag/AgCl electrode served as the reference, and a platinum wire served as the counter electrode. Prior to testing, samples were held at open-circuit potential (OCP) for 2 h to stabilize the system and determine the E_ocp_ value. Subsequently, anodic polarization curves of the materials were recorded using the potentiodynamic method. Samples were polarized from a potential 250 mV below E_ocp_ up to 500 mV. A scan rate of 0.2 mV/s was applied within ±250 mV of E_ocp_, followed by 1 mV/s up to 500 mV. From the resulting polarization curves, corrosion current densities (i_corr_) and corrosion potentials (E_corr_) were determined using Tafel extrapolation, while polarization resistance (R_pol_) values were derived using the Stern method [48]. All measurements were performed inside a Faraday cage, with at least three repetitions for each variant. The OCP results are shown as average values in the plots, whereas representative curves are provided for the potentiodynamic tests. After corrosion resistance tests, samples were examined under a Nikon Eclipse LV150N metallographic microscope (Nikon Instruments, Melville, NY, USA).

The quality of the adhesive bond between the coating and substrate was evaluated according to the Verein Deutscher Ingenieure (VDI) 3198 standard [49,50]. For each sample, three indentations were created at least 5 mm apart using a Rockwell diamond conical indenter on a Zwick/Roell ZHR 4150LK hardness tester (ZwickRoell, Ulm, Germany), applying a main load of 1500 N for approximately 10 s. After adhesion tests, samples were examined using a Nikon Eclipse LV150N metallographic microscope and a ThermoFisher Scientific Axia ChemiSEM HiVac 1247445 scanning electron microscope (Waltham, MA, USA). Based on the micrographs, the observed types of damage after indentation were described and assigned to categories defined by the VDI 3198 standard.

## 3. Results and Discussion

### 3.1. Open-Circuit Potential

Potentiodynamic corrosion resistance tests were performed on ground 34CrAlNi steel samples with developed surface topography in both a reference 3.5% NaCl solution and a solution simulating mine waters, representing environments with which the deposited coatings may come into contact during actual operation.

The open-circuit potential (OCP) in both electrolytes was most negative for the uncoated sample, stabilizing after 2 h at approximately −615 mV in both NaCl solution and mine water. Although DLC-coated samples initially exhibited significantly different E_ocp_ values, all stabilized over time within the range of −530 to −550 mV. In both environments, the coating deposited in a hydrogen-rich atmosphere (1:4:4) consistently demonstrated the highest E_ocp_ values.

**Figure 1:**
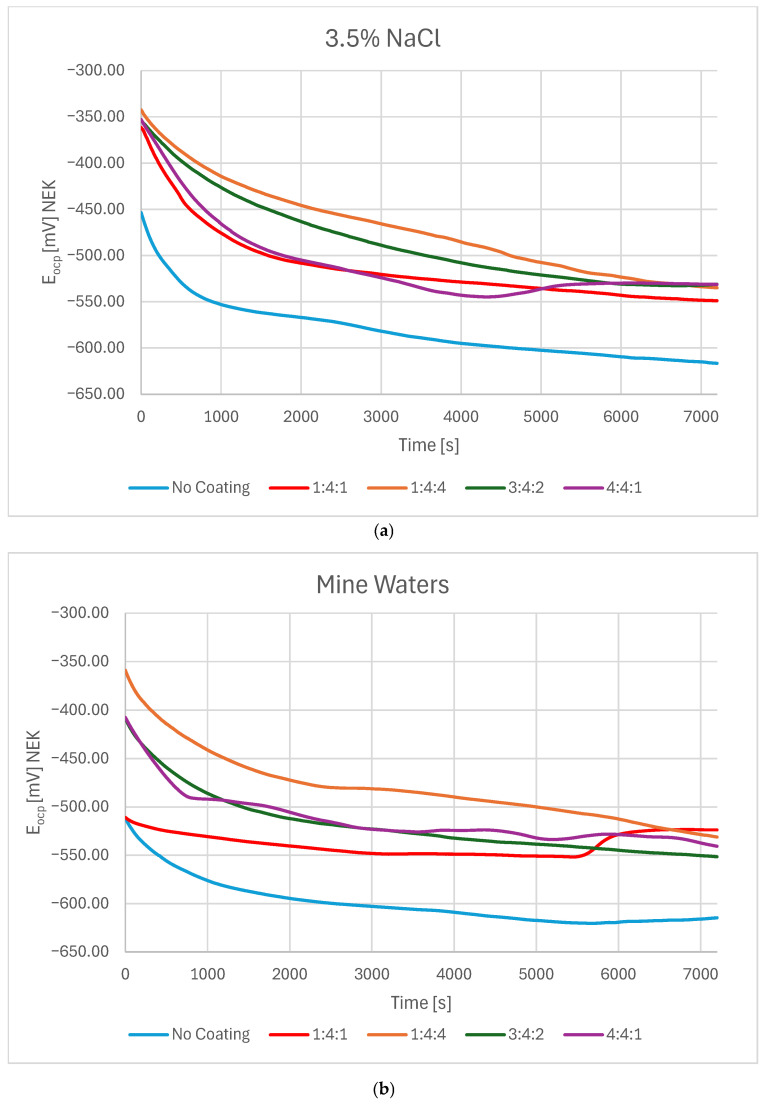
OCP change in time in (**a**) a 3.5% NaCl solution and (**b**) mine waters for carbon coatings produced under different reactive atmosphere compositions in the RFCVD process.

In 3.5% NaCl solution, all samples—including the uncoated reference—stabilized near the final potential after an initial rapid decrease, most likely associated with passivation of dangling bonds within the coating. The 1:4:1 and 4:4:1 coatings exhibited a sharper potential drop followed by greater stability (Figure 1a), which may indicate passivation of dangling bonds and non-hydrogenated carbon atoms with free valences. This behavior is consistent with the lower hydrogen content in these atmospheres, leading to a greater concentration of such bonds on the surface. By contrast, the 1:4:4 and 3:4:2 coatings reached the final potential through a more linear decrease. Similar trends were observed in mine water. However, in this environment, the 1:4:1 sample reached its target potential almost immediately, then maintained it following a gradual decrease and subsequent increase. The differences OCP observed for the DLC coatings can be attributed to variations in their microstructure and chemical composition, which depend on the deposition conditions. A higher sp^2^/sp^3^ ratio, hydrogen and hydrogen incorporation, or the presence of defects may influence the electrochemical stability of the passive surface film. Coatings with a denser and more homogeneous structure are expected to provide a more stable and noble OCP, as they better prevent electrolyte penetration and substrate interaction. The OCP values were slightly lower in mine water compared with NaCl solution (Figure 1b). This might be partially influenced by NO_3_^−^ presence, which creates the possibility of nitrate reduction reaction and can slightly lower the OCP in mine waters, and also due to differences in adsorption of ions in mixed-electrolyte solutions. For all coated samples, OCP was approximately 75 mV higher than for the uncoated substrate, stabilizing between −530 and −550 mV in both test solutions.

### 3.2. Potentiodynamic Measurements

**Figure 2:**
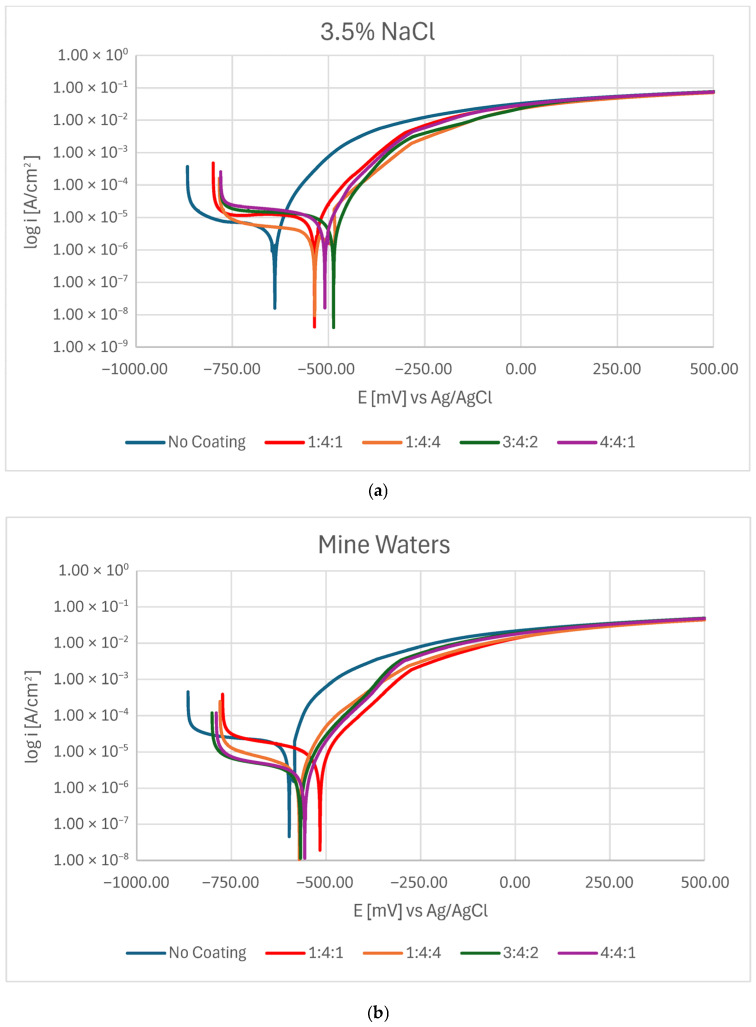
Potentiodynamic curves for carbon coatings produced under different gas proportions in the RFCVD process when tested in (**a**) a 3.5% NaCl solution and (**b**) mine waters.

**Figure 3:**
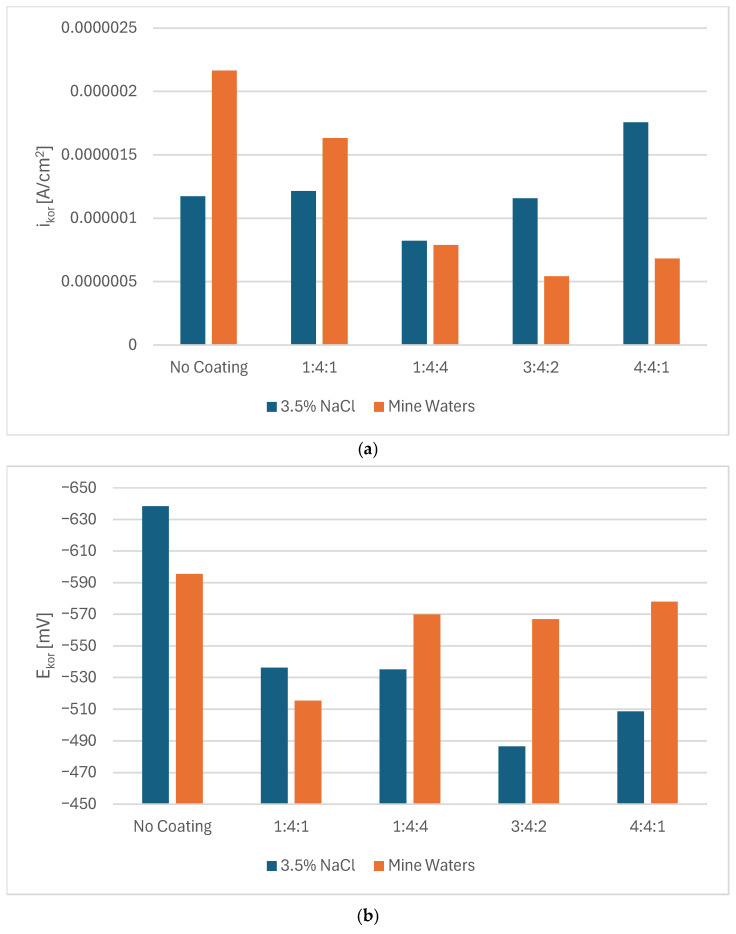

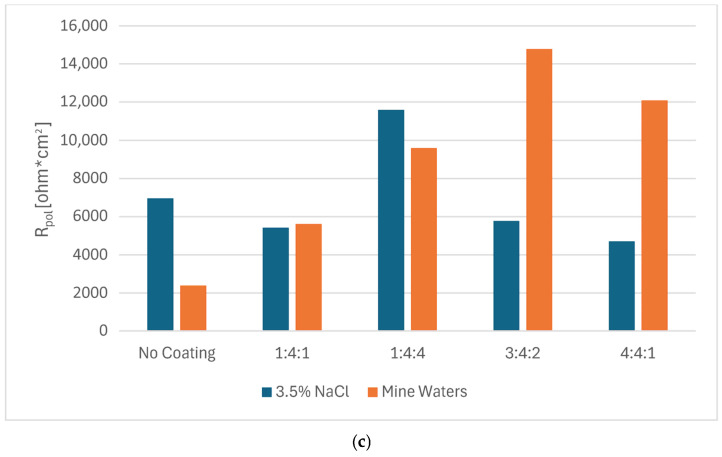
Values of corrosion current density (**a**), corrosion potential (**b**), and polarization resistance (**c**) for carbon coatings produced under different gas proportions in the RFCVD process in 3.5% NaCl solutions and mine waters.

Potentiodynamic measurements confirmed that in every case, the presence of a carbon coating improved the corrosion resistance of steel. Nevertheless, depending on the corrosive environment, coatings of different chemical compositions proved more advantageous (Figure 2a,b). In 3.5% NaCl solution, the 3:4:2 coating exhibited the highest corrosion potential, whereas the hydrogen-rich 1:4:4 coating demonstrated the lowest corrosion current density as well as the highest polarization resistance. In mine water (orange bars), the lowest E_corr_ was observed for the 1:4:1 coating, but the lowest I_corr_ (and thus highest R_pol_) was obtained for the 3:4:2 coating (Figure 3).

The slight shift in corrosion behavior between the 3.5% NaCl solution and mine waters might be caused by the influence of other ions in the solution. Without the high sulfate/Ca^2+^/NO_3_^−^ pool there is less competing adsorption/precipitation, so chloride can more freely concentrate at defects and drive local attack; this tends to raise I_cor_ and lower R_pol_ for coatings that are penetrated. Lower I_cor_ and higher R_pol_ for coatings with higher N and H content might be due to a combination of: (a) competitive sulfate adsorption and possible formation of Ca-bearing precipitates in coating defects (blocking active sites that might be more numerous in coatings with more dopants); and (b) the coating’s superior adhesion/barrier properties, which prevent aggressive ion transport into the metal/coating interface. It is, however, unclear why certain corrosion parameters rise while others fall for different coatings’ compositions as they do not show clear and consistent patterns. Chemical interactions between the dopants and different ions present in the solution might be complex and coincide with the thickness and adhesion of the coatings. As such, to assess the corrosion performance of amorphous carbon coatings in their target conditions, it might be insufficient to measure their performance only in model solutions such as 3.5% NaCl.

Although the differences between the corrosion parameters of the various carbon coatings were relatively small, in all cases they suggested at least partial improvement compared with the untreated martensitic substrate.

### 3.3. Corrosion Products

**Figure 4:**
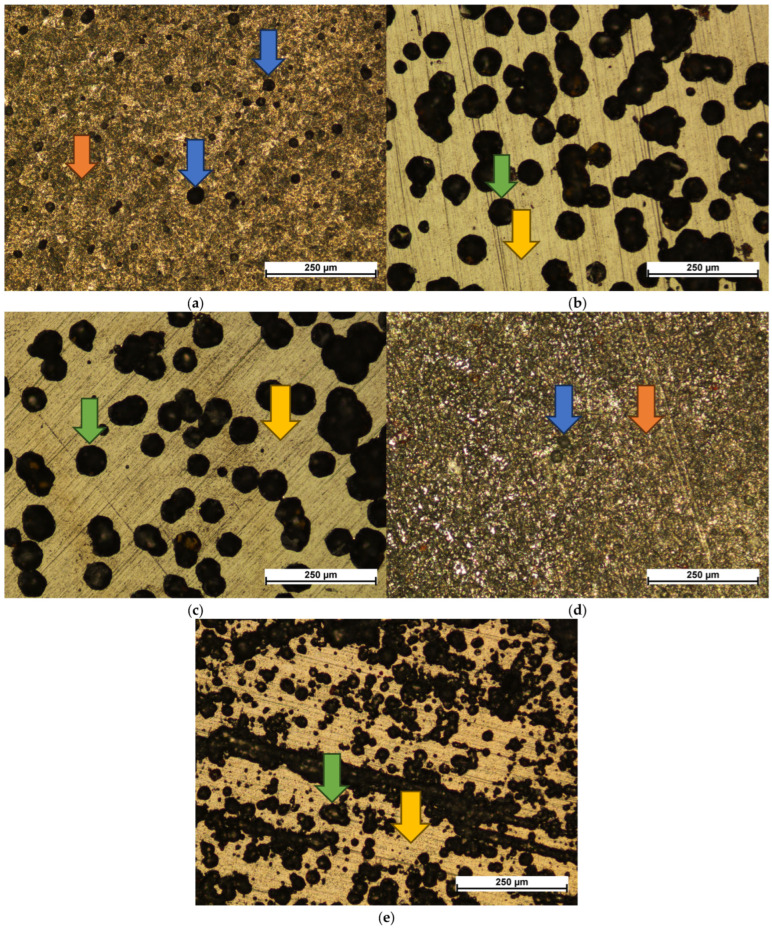
Corrosion damage observed on uncoated (**a**) and carbon-coated substrates in different atmospheric compositions: 1:4:1 (**b**), 1:4:4 (**c**), 4:4:1 (**d**), and 3:4:2 (**e**). The blue arrows indicate pits; orange arrows—uniform corrosion; green arrows—delamination of the coatings with pitting corrosion on the exposed surface; yellow arrows—the undamaged coatings.

Surface images of samples after corrosion testing in the 3.5% NaCl solution revealed the characteristic damage modes associated with each coating (Figure 4). The uncoated reference sample exhibited clear uniform corrosion with visible pits, which is expected for steel of this chemical composition (Figure 4a). For nearly all coated samples (except 4:4:1), no corrosion traces were observed in areas where the carbon coating remained intact (Figure 4b,c,e). In the case of the 4:4:1 coating, uniform etching of the steel surface occurred (Figure 4d), possibly due to differences in chemical composition, e.g., higher oxygen content, or due to easier spallation of the coating after its initial cracking.

The 1:4:1 and 1:4:4 coatings displayed similar damage patterns—circular delamination of the coating, within which pitting corrosion developed (Figure 4b,c). While the overall damage mechanism did not change, fewer instances of such delamination were observed on the 1:4:4 sample, possibly due to the greater coating thickness. The 3:4:2 coating exhibited a similar type of damage, but the spallations were significantly finer and concentrated around topographic features such as ridge lines created during sample preparation (Figure 4e). This behavior is likely influenced by both its lower thickness and high adhesion, which limited coating failure to small localized areas.

The obtained corrosion test results are consistent with the literature data, where carbon coatings enhanced the corrosion resistance of steel in a 0.1 M NaCl environment [27]. The corrosion current densities are comparable with those reported in other studies [27]; however, the improvement in properties is less pronounced due to the inherently higher corrosion resistance of the substrate. Another factor influencing the corrosion resistance of the deposited coatings may be their relatively small thickness. Since corrosion resistance increases with thickness [23], it can be expected that sample 1:4:4 would perform best. This trend is indeed confirmed by the lowest corrosion current density and the highest polarization resistance observed for this sample in the 3.5% NaCl solution.

As in previous meta-analyses [18], no clear correlation was observed between the corrosion behavior of the coatings and the sp^3^ bond content. In contrast to the reduction in corrosion resistance reported after the introduction of dopants [11,30,31], no such effect was observed here. This may be attributed to the simultaneous increase in sp^3^ bond content during the deposition of coatings co-doped with nitrogen and hydrogen. Moreover, higher corrosion resistance has been reported for thinner coatings [51], which according to other studies also exhibit superior adhesion to the steel substrate [47]. Ultimately, adhesion appears to be a more decisive parameter than dopant content in the studied samples—or more precisely, the influence of dopants on adhesion seems to outweigh their impact on electrochemical parameters.

Since the tests were intentionally conducted on a surface with developed roughness, simulating the actual texture of industrial components, this may have contributed to the reduced corrosion resistance of the coatings. Such surfaces inherently contain more heterogeneities, which act as sites of elevated energy and are therefore more prone to corrosion [27]. Although the use of RFCVD combined with preliminary cathodic sputtering allows for partial surface cleaning, it cannot be assumed that this process eliminates all contaminants from a surface pre-treated by etching.

In the 3.5% NaCl solution, maximizing the coating thickness proved to be the most effective strategy for improving corrosion resistance. This can be explained by the coating’s inherent resistance to the corrosive medium, combined with strong adhesion to the substrate, which limited corrosion damage within areas where the coating was penetrated during potentiodynamic measurements.

The influence of dopants on corrosion performance in the NaCl solution was not significant. However, in the simulated mine water environment, sample 3:4:2—with the highest nitrogen content—showed the lowest corrosion current density and highest polarization resistance, indicating an improvement in properties due to co-doping with hydrogen and nitrogen. Since the introduction of dopants usually decreases the corrosion resistance of carbon coatings [11,30,31], this effect should rather be attributed to enhanced adhesion of the coating to the substrate. This effect has also been reported in the literature, although primarily in cases where an interlayer [9] or softening dopants such as fluorine [29] were introduced—the latter role being fulfilled here by nitrogen [34,41,47,52].

### 3.4. Adhesion Quallity

**Figure 5:**
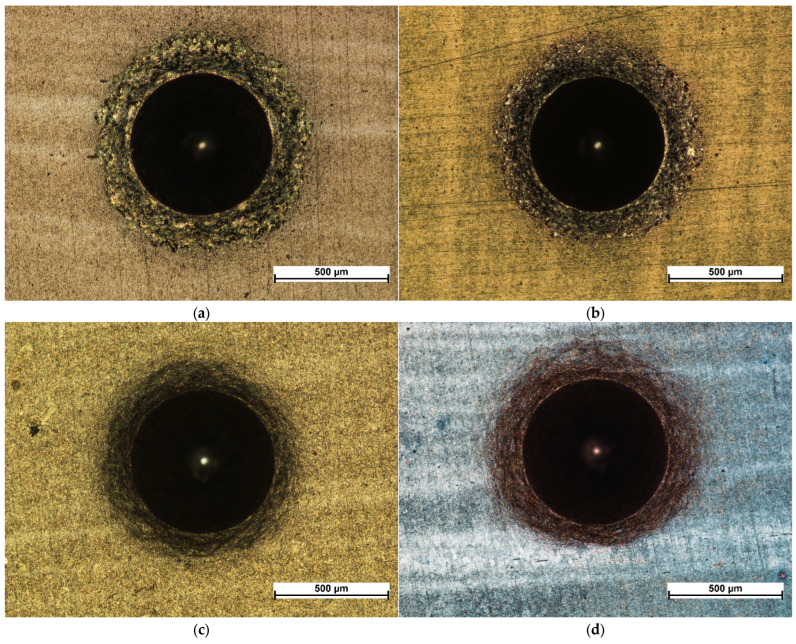
Indents created according to VDI 3198 for coatings produced under the atmosphere compositions of: 1:4:1 (**a**), 1:4:4 (**b**), 4:4:1 (**c**), and 3:4:2 (**d**) under a metallographic microscope.

**Figure 6:**
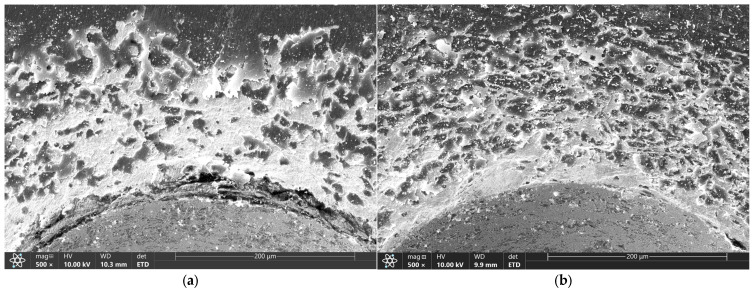

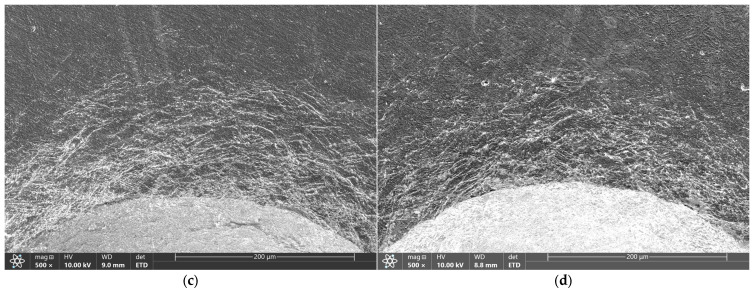
Indents created according to VDI 3198 for coatings produced under atmospheric compositions of 1:4:1 (**a**), 1:4:4 (**b**), 4:4:1 (**c**), and 3:4:2 (**d**), as observed under an SEM.

Microscopic images of the sample surfaces in the regions of indentations prepared in accordance with the VDI 3198 standard demonstrate that all studied coatings exhibit very strong adhesion to the substrate. For coatings deposited in 3:4:2 and 4:4:1 atmospheres, only a dense network of fine cracks was observed around the indentation edges, corresponding to adhesion category HF2 (Figure 5c,d and Figure 6c,d). In contrast, coatings deposited in 1:4:1 and 1:4:4 atmospheres showed chipping and delamination of varying size, which still falls within the acceptable damage range but corresponds to categories HF3 (1:4:4) and HF4 (1:4:1) (Figure 5a,b and Figure 6a,b) [49,50]. These results confirm that their adhesion is inferior compared with coatings prepared in 3:4:2 and 4:4:1 atmospheres, which is consistent with findings from scratch test studies performed on coatings deposited under similar conditions [47].

The obtained results correlate with those from scratch tests conducted on coatings produced under the same conditions, where coatings deposited in 4:4:1 and 3:4:2 atmospheres exhibited the highest critical loads for complete delamination (13.9 and 15.7 N, respectively), while for the 1:4:1 and 1:4:4 atmospheres the values were 8 and 9 N, respectively [47]. Such a correlation between increased corrosion resistance and enhanced adhesion of carbon coatings has also been reported in the literature [24]. At the same time, it can be observed that the corrosion damage on samples 1:4:1 and 1:4:4 is more extensive but less numerous, whereas in the case of 3:4:2 and 4:4:1, the damage is finer yet densely distributed across the surface (Figure 5). These observations are likely interrelated: improved adhesion of the coating to the substrate hinders the propagation of corrosion-induced defects, requiring failure of the coating at multiple sites.

The chemical composition of coatings deposited under analogous conditions showed that the 3:4:2 atmosphere yielded the highest sp^3^ bond content (over 30%, compared with 21–23% for the other atmospheres) [47]. However, this did not result in markedly different corrosion behavior compared with coatings of similar adhesion. This observation is consistent with comparative studies, which did not reveal a clear correlation between the corrosion resistance of carbon coatings and the sp^2^/sp^3^ bond ratio [18]. On this basis, it can be concluded that the adhesion of carbon coatings exerts a far greater influence on corrosion resistance than their chemical composition or the proportion of specific bond types. The latter affect the corrosion behavior of carbon coatings primarily through their impact on adhesion.

## 4. Conclusions

- Carbon coatings significantly enhance the corrosion resistance of 34CrAlNi steel substrates.
- The most favorable corrosion resistance parameters in an environment simulating actual service conditions were achieved by the 3:4:2 coating.
- The decisive factors influencing corrosion behavior were coating thickness and adhesion to the substrate, which governed electrochemical parameters and the nature of damage to a greater extent than chemical composition.
- Increasing the hydrogen and nitrogen content in carbon coatings improves their corrosion resistance by enhancing coating adhesion to the substrate.

## Figures and Tables

**Table 1:** Chemical composition of 34CrAlNi steel according to the manufacturer data.

Element Content [%]							
C	Si	Mn	P	S	Cr	Ni	Al
0.3–0.37	<0.4	0.4–0.7	<0.025	<0.035	1.5–1.8	0.85–1.15	0.8–1.2

**Table 2:** Chemical composition of the mine water.

Content [mg/L]				
NO_3_^−^	Cl^−^	SO_4_^2−^	Ca^2+^	HCO_3_^−^
24,085	24,014	63,345	1000	80

## Data Availability

The original contributions presented in this study are included in the article. Further inquiries can be directed to the corresponding author.
